# T-cell epitope polymorphisms of the *Plasmodium falciparum *circumsporozoite protein among field isolates from Sierra Leone: age-dependent haplotype distribution?

**DOI:** 10.1186/1475-2875-8-120

**Published:** 2009-06-05

**Authors:** Amadu Jalloh, Muctarr Jalloh, Hiroyuki Matsuoka

**Affiliations:** 1 Division of Medical Zoology, Department of Infection and Immunity, Jichi Medical University, Yakushiji 3311-1, Shimotsuke, Tochigi 329-0498, Japan; 2 College of Medicine and Allied Health Sciences, University of Sierra Leone, Private Mail Bag, Freetown, Sierra Leone; 3 Princess Christian Maternity and Children's Hospital, Freetown, Sierra Leone

## Abstract

**Background:**

In the context of the development of a successful malaria vaccine, understanding the polymorphisms exhibited by malaria antigens in natural parasite populations is crucial for proper vaccine design. Recent observations have indicated that sequence polymorphisms in the C-terminal T-cell epitopes of the *Plasmodium falciparum *circumsporozoite protein (Pf*csp*) are rather low and apparently stable in low endemic areas. This study sought to assess the pattern in a malaria endemic setting in Africa, using samples from Freetown, Sierra Leone.

**Methods:**

Filter-paper blood samples were collected from subjects at a teaching hospital in Freetown during September–October 2006 and in April–May 2007. The C-terminal portion of the Pf*csp *gene spanning the Th2R and Th3R epitopes was amplified and directly sequenced; sequences were analysed with subject parameters and polymorphism patterns in Freetown were compared to that in other malaria endemic areas.

**Results and Discussion:**

Overall, the genetic diversity in Freetown was high. From a total of 99 sequences, 42 haplotypes were identified with at least three accounting for 44.4% (44/99): the 3D7-type (19.2%), a novel type, P-01 (17.2%), and E12 (8.1%). Interestingly, all were unique to the African sub-region and there appeared to be predilection for certain haplotypes to distribute in certain age-groups: the 3D7 type was detected mainly in hospitalized children under 15 years of age, while the P-01 type was common in adult antenatal females (Pearson Chi-square = 48.750, degrees of freedom = 34, *P *= 0.049). In contrast, the single-haplotype predominance (proportion > 50%) pattern previously identified in Asia was not detected in Freetown.

**Conclusion:**

Haplotype distribution of the T-cell epitopes of Pf*csp *in Freetown appeared to vary with age in the study population, and the polymorphism patterns were similar to that observed in neighbouring Gambia, but differed significantly at the sequence level from that observed in Asia. The findings further emphasize the role of local factors in generating polymorphisms in the T-cell epitopes of the *P. falciparum *circumsporozoite protein.

## Background

Every year malaria kills more than a million people worldwide [1] and the need for an effective vaccine to boost control efforts cannot be over-emphasized. In the context of the development of a successful malaria vaccine, a major potential obstacle is the issue of genetic polymorphisms exhibited by malaria antigens. While an understanding of these polymorphisms in natural plasmodium populations is not only crucial for proper vaccine design, deployment and evaluation, such data may also provide invaluable insights into host-parasite interaction(s). The *Plasmodium falciparum *circumsporozoite protein (Pf-CSP) is the most abundant protein on the sporozoite's surface and has a sequence comprising a central repeat that is flanked by polymorphic N-terminal and C-terminal non-repeat regions. The Pf-CSP antigen is the primary component of the RTS,S [2], which is one of the leading malaria vaccine candidates, and the only one to have shown moderate, but promising, results during Phase II trials in adults in The Gambia [3] and children in Mozambique [4]. Plans for Phase III trials are currently underway in 10 African nations [5]. Although field data on malaria antigen polymorphisms generally have been rather scanty, there is now an improving trend partly due to increased training of and collaboration with researchers in endemic nations.

With regards to the Pf*csp *gene, two distinct polymorphisms have been described: polymorphism in the number of NANP repeats and single nucleotide polymorphisms (SNPs) in non-repeat regions. Although polymorphisms in the number of NANP repeats can be extensive even in low endemic areas [6], it has been documented that SNPs in the non-repeat regions (Th2R/Th3R epitopes) in such settings, e.g. Southeast Asia, are stable for an observation period of at least five years in Vietnam [7], or 10 years in Thailand [8]. Moreover, the overall scenario suggests that Asian parasites (with respect to the Th2R/Th3R regions) are somewhat "clonal", with one majority sequence predominating, probably reflecting distinct differences between parasites from Asia (lower endemicity) and Africa (higher endemicity). However, samples examined from Africa at the population level tended to be relatively small and with variation in sampling time-points. These findings prompted the investigation of the diversity of Pf*csp *gene in a typical African malaria-endemic setting in Sierra Leone. With no previous data on this important topic in the country, the main aim herein was to collect baseline sequence data and to characterize sequence polymorphisms of the T-cell epitopes of Pf-CSP among field isolates distributing in the capital, Freetown, with an ultimate aim of acquiring insight into host-parasite interaction(s). In addition, this study examined whether there was any significant difference in polymorphism patterns between African and Asian parasites. Results confirmed marked sequence diversity of African isolates as compared to Asia, with at least three major haplotypes in Freetown. Moreover, haplotype distribution in this endemic area appeared to vary with age, and presumably immunity status. Implications of findings are discussed with regards to malaria vaccine design and local malaria epidemiology.

## Methods

### Study area

This study was conducted at the Princess Christian Maternity and Children's Hospital (PCMH), as part of a study on G6PD deficiency polymorphisms, which has been described in [9]. The PCMH is located in the East End of Freetown, where it is surrounded by overcrowded residential and market areas. As the name implies, the hospital not only plays a vital role in maternity medicine, but also serves as one of the main national paediatric centers in the country. Malaria transmission in Freetown is perennial with apparent fluctuations in intensity during the rainy (May–October) and dry (November–April) seasons. Although there have been no recent malaria-related entomologic studies in the capital, earlier studies have identified *Anopheles gambiae*, *Anopheles funestus*, *Anopheles melas*, and *Anopheles nili *as efficient local malaria vectors, with *P. falciparum *being the dominant parasite species (reviewed in [10]). Previous research [10] also identified the capital as fluctuating between that of a meso- and hyper-endemic area, but studies are urgently required to properly assess the current situation. Despite the long history of malaria control activities in Sierra Leone, the disease is still a major public health problem and accounts for about 40% of outpatient consultations in the country. Presumptive diagnosis followed by self-treatment is not uncommon, and is thought to contribute greatly to the widespread drug resistance recently documented in the country, warranting a recent shift in national malaria treatment policy [11].

### Sample collection

Hospital-based surveys were conducted in mid-September to mid-October 2006 and in mid-April to mid-May 2007. Participants in this study comprised two main groups of individuals residing in Freetown: (i) symptomatic children visiting and/or admitted at the PCMH, as well as (ii) antenatal out-patients visiting the PCMH microbiology laboratory for routine blood tests (e.g. malaria microscopy, differential cell count, haemoglobin levels etc.). Patients were introduced to the study and those consenting were screened for malaria via microscopy, and filter paper blood samples were collected for each subject. Filter paper blood spots were air-dried, sealed in tiny plastic bags and stored at room temperature (25–31°C), until later molecular analysis at Jichi Medical University, Tochigi, Japan. Prior to blood donation, subjects were interviewed and basic demographic information for each participant were recorded including age, sex, ethnicity, and whether they were admitted at the hospital (i.e. in-patients) or only visiting (out-patients). Falciparum parasitaemia (parasite density) was determined for each subject by local microscopists, but due to technical limitations encountered in the field parasite density data was not utilized further.

### DNA extraction and sequencing

Genomic DNA was extracted from randomly selected filter paper blood samples using the GFX kit (GE Healthcare, UK), regardless of microscopy results. The C-terminal portion spanning the T-cell epitope regions of the gene of *P. falciparum csp *was amplified by a semi-nested PCR using primers and cycle conditions described in [12]. In brief, the first PCR used primers 5'-aatcaaggtaatggacaagg-3' (CSo101) and 5'-ctaattaaggaacaagaagg-3 (CSo102); and 1 μl of the PCR product was used in a second round PCR using primers CSo101 and a second reverse primer, 5'-ggaacaagaaggataatacc-3' (CSo104) in a 20 μl reaction mixture. Amplified gene fragments were resolved on 2% agarose gels following electrophoresis and visualized by ultraviolet (UV) light. The fragments of positive samples were excised using FUNA Gel pipette tips (Funakoshi, Tokyo, Japan), and diluted with 40–100 μl deionized distilled water depending on band intensity. Five microlitres of diluted DNA was used as template for cycle sequencing in a 10 μl reaction and the reverse primer was used to read the sequences for each fragment on an ABI 310 Genetic Analyzer. In principle, sequences were read once per sample using the second reverse primer (CSo104) but when a new mutation was encountered or when two electrophoregram peaks overlapped at a known mutation site, sequencing was repeated with another independent sample using the forward primer (CSo101).

### Sequence alignment and statistical analyses

Sequences were aligned using MEGA4 software [13] and manually edited, with the sequence of the 7G8 strain from Brazil [14] serving as reference. Each *csp *sequence was treated as an independent entity, coded and statistical analysis was conducted using the SPSS 10.0 software for Windows. The 2-sided Pearson Chi-square test of independence for categorical variables was used to compare the respective frequencies of haplotypes with regards to subject parameters including age, ethnicity, sex, and "status" (i.e. whether hospitalized or not), as well as time of sample collection. This was done in an attempt to examine the hypothesis whether haplotype distribution was associated with age (and presumably immunity status) or any of the other parameters described above. Statistical significance was set at the 5% level (*P *= 0.05).

In an effort to examine geographical diversity patterns with other parasite populations, the C-terminal portion of recently published Pf*csp *sequences from Vietnam [7] (*n *= 142), from Iran (*n *= 90) described in [15], and from one cohort (*n *= 44) of field isolates from neighbouring Gambia [16] were analysed. Commonly used intra-population and inter-population genetic parameters were estimated using DnaSP 4.5 software [17] and compared between African (The Gambia and Sierra Leone) and Asian sequences (Vietnam and Iran). Parameters investigated include nucleotide diversity, *pi *(π) [18], which is the average number of substitutions between any two sequences, and haplotype (gene) diversity values [18]. In addition, the Wright's Fixation statistic (F_ST_) [19], which measures genetic differentiation between sub-populations, and Nei's parameters [18] including the average number of nucleotide differences per site (Dxy), net nucleotide substitution per site (Da), and average proportions of nucleotide substitutions per site (Kxy) between populations, were also estimated.

This study was approved by the Ethical Committees of the PCMH, University of Sierra Leone and Jichi Medical University, Tochigi, Japan, and the partial Pf*csp *sequences obtained herein have been submitted to GenBank with accession numbers GQ119637–GQ119678.

## Results

### Overview of data

Table 1 describes the demographic characteristics of all study subjects in the PCMH 2006 study-group who provided blood samples, as well as the number of DNA samples successfully sequenced and analysed. For analytic purposes, subjects from this PCMH 2006 study-group were categorised into three main age groups: (i) below five years of age, (ii) 5–14 years of age, and (iii) 15 years and older. Of 164 filter paper blood samples, DNA was isolated from 102 samples (i.e. isolates) of which 81 showed bands (positive samples) following PCR amplification for the target Pf*csp *gene fragment of 321 base pairs (bp), and these were successfully sequenced. Sixty-six sequences were of clear electrophoregram peaks showing no or low (<1/3) background overlap of the dominant peak. These were referred to as "single dominant" sequences and coded as "single-clone" infections. Six samples revealed sequences with electrophoregram peaks overlapping at one known mutation site for which both possible amino acid sequences were deduced from the nucleotide sequence (total *n *= 6 × 2). Nine samples showed chromatogram peak overlap at two or more known mutation sites (termed "mixed-clone" infections), and were omitted in the analysis presented here.

**Table 1:** Summary characteristics of the PCMH 2006 study subjects (N = 164)

	Age (in years)		Number of subjects		Total^a^
			
Sex	Group	Mean ± SD	Inpatient	Outpatient	N (n)
Female	<5 yr	2.1 ± 1.4	16 (4)	14 (5)	30 (9)
	5–14 yr	8.5 ± 3.0	3 (1)	14 (3)	17 (4)
	≥ 15 yr	25.6 ± 6.7	3 (0)	67 (18)	70 (18)
Subtotal		17.1 ± 11.9	22 (5)	95 (26)	117 (31)
Male	<5 yr	1.8 ± 1.3	9 (7)	14 (14)	23 (21)
	5–14 yr	8.5 ± 2.8	8 (6)	8 (8)	16 (14)
	≥ 15 yr	26.5 ± 7.5	0 (0)	8 (6)	8 (6)
Subtotal		8.3 ± 9.5	17 (13)	30 (28)	47 (41)

^a ^N represent the total number of blood sample donors and numbers in parentheses indicate subjects whose DNA samples were successfully sequenced and analyzed (n = 72)

**Figure 1:**
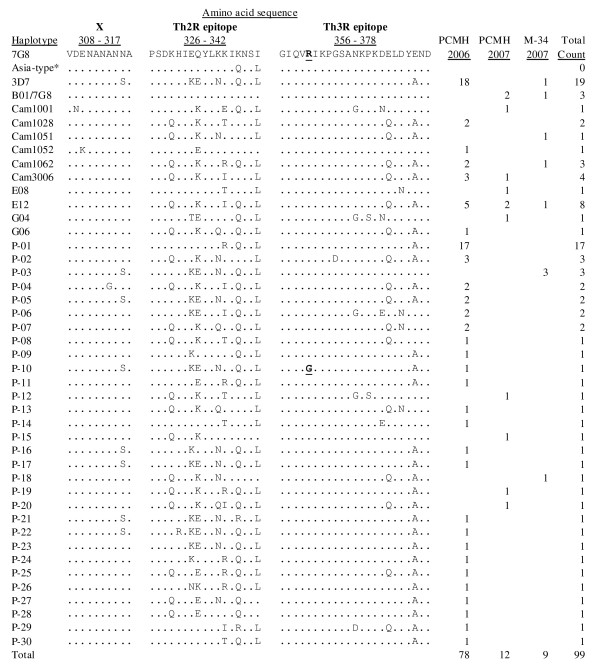
**Sequences of *P. falciparum csp *haplotypes found among field isolates from two hospitals (PCMH and M-34) in Freetown, Sierra Leone, during September–October 2006 and April–May 2007**. Note that only variable sites immediately after the central repeats (labeled X i.e. amino residues 308 – 317), and the regions spanning the Th2R (amino residues 326 – 342) and Th3R (amino residues 356 – 378) in the C-terminal portion of the gene are shown. Amino residue numbering is with reference to the 7G8 sequence and haplotypes labeled P-01 ~ P-30 are unique to this study. The P-17 haplotype differs from the 3D7 sequence by two silent nucleotide mutations but otherwise has an identical amino acid sequence. As shown, the "Asia-type*" refers to the Th2R*5/Th3R*1 haplotype previously shown to be predominant in Asia, but was not detected in this study.

Among blood samples collected during the April–May 2007 survey, an additional set collected from the Thirty Fourth Military Hospital (M-34) at Wilberforce (located in the Western Area of Freetown, about 6 Km from the PCMH) were also analysed. Of these 2007 samples, 12 of 47 (25.5%) from the PCMH and nine of 30 (30.0%) from M-34 amplified and were sequenced, and all revealed "single-dominant" sequences (Figure 1). There was a significant difference (Chi-square = 3.84, degree of freedom, d.f. = 1, *P *< 0.0001) in the number of PCR-positive samples between isolates collected in September–October 2006 i.e. during the rainy season (81/102 or 79.4%) and in April–May 2007 i.e. the dry season (21/77 or 27.2%).

### Haplotype distribution with age, sex and other parameters

Pooling the data and analysis of the whole set of 99 C-terminal *csp *sequences (including the six double-clone infections) from Freetown identified a total of 42 unique haplotypes for which the respective proportions are depicted in Figure 1. Three major haplotypes were detected: the 3D7-type (19.2%) which is identical to the RTS,S vaccine sequence, a previously undescribed haplotype, P-01 (17.2%), and E-12 (8.1%) a sequence that has been reported from neighbouring Gambia. A majority of the remaining haplotypes comprised mostly minority alleles and almost all were unique to the West African sub-region at the nucleotide level. Consistent with previous studies, almost all mutations observed (except for haplotype P-17) were non-synonymous, and clustered in the T-cell epitope regions (Th2R, Th3R) of the gene, although a few sequences had mutations in the region preceding the Th2R (labeled X in Figure 1). Moreover, there was an Arg > Gly substitution (haplotype P-10 in Figure 1) in the highly conserved region II i.e. a region sandwiched between the Th2R and Th3R, which has been implicated in hepatocyte binding [20]. Interestingly, most of the observed haplotypes usually differed from one another by one non-silent point mutation in the whole C-terminal region. For example, the P-10 haplotype differed from the 3D7 sequence only by the amino residue substitution at position 360 in Figure 1, while the P-01 isolate that dominated in antenatal out-patients differed from the P-30 isolate only by the R>T substitution at residue position 337. Haplotypes E12 and Cam3006 differed by one mutation in Th2R, whereas 3D7 and P-03 differed by a mutation in the Th3R. A similar trend was observed for other haplotypes, tempting the speculation that some form of recombination events might be occurring in these C-terminal T-cell epitope regions of CSP in natural parasite populations.

**Figure 2:**
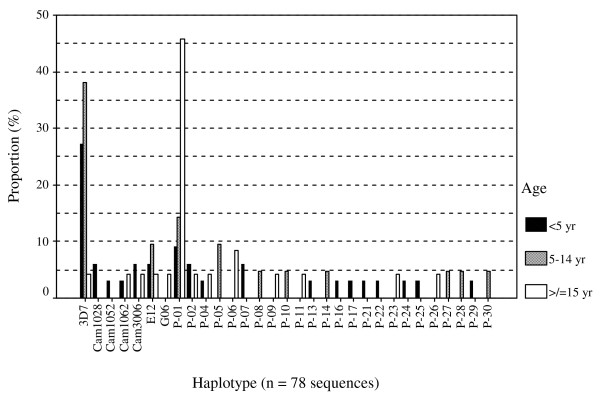
**Distribution of *csp *haplotypes by age category among the PCMH 2006 sequences for which n = 33, 21, and 24 for the 3 respective age groups**: (i) less than 5 years (black bars) of age (ii) between 5 and 14 years (grey bars) and (iii) 15 years or older (open bars). Note that all sequences from single-clone as well as the six double-clone infections were used in this analysis. The 3D7 haplotype was found more in children aged less than 15 years while the P-01 allele was more prevalent in adult females, who were mostly antenatal follow-up subjects.

Preliminary analysis for the whole set of 99 sequences revealed a non-significant (Pearson Chi-square value of 93.219, degrees of freedom = 82, *P *= 0.187), yet unexpected haplotype distribution pattern with respect to age. As shown in Figure 2, the 3D7 and a previously undescribed haplotype (P-01) appeared to predominate in different age groups, in which the former was associated with mainly children under 15 years and the latter with adult women. Subsequent sub-group analysis of the 2006 PCMH data (using all sequences including single as well as the six double-clone infections) by age, sex, ethnicity and patient status (i.e. in-patient or out-patient) revealed a significant, albeit weak, association only for the out-patient females (Pearson Chi-square value of 48.750, d.f. = 34, *P *= 0.049), who were mainly pregnant women visiting for regular antenatal follow-up. However, repeating the analysis with one sequence per isolate (i.e. using a randomly selected sequence from the six double-clone infected samples) reduced the p-value slightly (Pearson Chi-square value of 46.944, d.f. 32, *P *= 0.043). The availability of fewer sequences in other sub-groups precluded further comprehensive analysis with respect to sampling time and place. Nonetheless, it appears that, in this African endemic setting, haplotype frequencies are likely to vary with time (e.g. season), as demonstrated between samples collected in 2006 (rainy season) and 2007 (dry season) in Figure 1, as well as by the proportion of PCR-positive samples in 2006 i.e. 79.4% (81/102) versus 27.3% (21/77) in 2007.

### Geographical distribution of *csp *haplotypes

Spatial polymorphism patterns were examined by comparing recently published sympatric (of parasites from the same locality at a given time) *csp *sequences obtained from hospital-based surveys in different countries [7,15,16]. These data were acquired through direct sequencing of isolates, allowing appropriate comparison to data presented here, and only the region (207 bp) spanning the Th2R and Th3R epitopes was used (i.e. spanning amino acid positions 326 – 394). Of 375 sequences 126 (33.6%) were of the predominant haplotype i.e. the Th2R*5/Th3R*1 [7,15,16] unique to Asia (Vietnam: 75/142 or 52.8% and Iran: 51/90 or 56.7%), but was not found in The Gambia nor in Sierra Leone (Additional File 1). Similarly, other common haplotypes in West Africa (3D7, P-01, E12) were not detected in Vietnam or in Iran, while the 7G8 sequence was found in all three countries except Vietnam. While this differential *csp*-haplotype distribution was statistically significant by Chi-square analysis using SPSS (Pearson Chi-square = 766.847, d.f. = 219, *P *< 0.00001), it is of interest to note that not finding a particular haplotype does not completely preclude its existence in the locality of interest but may simply suggest it might be of low frequency. Another striking feature was that all mutations were almost always restricted to the same positions in the T-cell epitope regions of the gene, regardless of parasite origin. What seemed important was the nature of the substituting amino acid, again suggesting strong functional constraints on the CSP molecule.

As depicted in Table 2, within-population genetic diversity estimates (π, haplotype diversity) reveal consistent patterns in all countries, with the Th2R epitope region being more polymorphic than the Th3R region. However, between-population genetic differentiation parameters (F_ST_, Dxy, Kxy and Da) indicate regional differences, with African parasites having similar indices than they are to Asian parasites. This data is probably reminiscent of differences in transmission intensities/dynamics between the respective geographic regions.

**Table 2:** Intra-population genetic diversity and inter-population genetic differentiation estimates of Asian and African parasites

(a) Intra-population genetic diversity parameters^a^						
	No. of	No. of	Haplotype	Nucleotide diversity per site (*π*)		
				
Population	sequences	haplotypes	diversity	Th2R	Th3R	All sites
Gambia	44	21	0.95137	0.07526	0.04343	0.02882
Sierra Leone	99	40	0.91899	0.06753	0.02926	0.02366
Vietnam	142	20	0.69693	0.02625	0.01420	0.00983
Iran	90	5	0.60275	0.01530	0.00632	0.00530
(b) Inter-population genetic differentiation parameters						
Population-1	Population-2		F_ST_^b^	Dxy^c^	Da^d^	Kxy^e^

Gambia	Sierra Leone		0.04403	0.02745	0.00121	5.68205
Gambia	Vietnam		0.24410	0.02557	0.00624	5.29209
Gambia	Iran		0.27967	0.02366	0.00662	4.89798
Sierra Leone	Vietnam		0.31434	0.02442	0.00768	5.05499
Sierra Leone	Iran		0.37256	0.02305	0.00859	4.77149
Vietnam	Iran		0.13221	0.00870	0.00115	1.80031

^a^Estimates are based on pair wise analyses of 375 sequences of the C-terminal portion (207 bp) of the CSP gene spanning the Th2R and Th3R using DnaSP4.5 (21)

^b^FST: Wright's Fixation index, which is a measure of genetic differentiation between populations (range from 0 to +1)

^c^Dxy: measures the average number of nucleotide differences per site between populations

^d^Da: measures the number of net nucleotide substitutions per site between populations

^e^Kxy: measures the average proportions of nucleotide substitutions per site between populations

## Discussion

Presented here is the first malaria molecular epidemiologic study in Sierra Leone on the genetic diversity of T-cell epitope regions in the C-terminal portion of the *P. falciparum *circumsporozoite protein among wild isolates circulating in the capital, Freetown. Contrary to previous data presented from moderate to low endemic settings of malaria [6-8,15], results of this study indicate that sequence polymorphisms in the same region of Pf*csp *(Th2R/Th3R epitopes) in an African setting are markedly diverse, but again with some degree of restriction. In addition, the single-haplotype predominance of the "Asia type" sequence (proportion > 50%) previously documented in Asia (Additional file 1) was not observed in this African setting. Instead three haplotypes accounted for the majority (44.4%) of the sequences i.e. 3D7 (19.2%), P-01 (17.2%) and E12 (8.1%), and with intriguing distribution patterns. The 3D7 allele was associated with hospitalized children in two age groups (<5 years of age and 5–15 years of age), but not with adults, while another previously undescribed haplotype (P-01) was significantly associated with visiting antenatal subjects (*P *= 0.049). Since most of these antenatal females were of mild or asymptomatic infection, this finding inevitably warrants the speculation whether distribution of *P. falciparum csp *haplotypes could be associated with disease severity i.e. does naturally-acquired immunity select for certain CSP variants in malaria-endemic areas? While the data presented here may in part support this notion, it may also be argued that this study may have suffered due to limited sample size (thus underpowered), or the findings may have been simply due to chance (borderline *P *values). Whatever the case, this haplotype distribution pattern in an African endemic area is important in that it adds insight into a relevant issue in vaccine design and evaluation i.e. could there indeed be significant differences of vaccine effects in *symptomatic *versus *asymptomatic *malaria individuals, particularly in the context of an African endemic setting where transmission is usually intense? This might be a major issue for consideration in future large-scale longitudinal studies on malaria antigen polymorphisms of vaccine antigens in other endemic nations.

Despite the documented polymorphisms in CSP, the mechanism(s) behind them are poorly understood. Proposed mechanisms include intragenic recombination and natural selection (human immune pressure- or mosquito-induced). In recent field trials of the most advanced CSP vaccine (RTS,S), it was demonstrated that vaccine-mediated immunity was not strain-specific [21], nor did the vaccine induce T-cell epitope selection [12]. A subsequent study using samples from Thailand further questioned the contribution of human immune pressure towards epitope diversity in the *csp *[8]. Thus it was suggested that this scenario could be due to a major contributory role of "mosquito-induced" selection by the different vector species transmitting malaria in the different geographic regions [8,15]. However, it appears that this "mosquito-induced" selection may not completely explain the observed differential haplotype distribution patterns in different endemic settings, as documented in this study. Moreover, although one study recently demonstrated that sterile protection is independent of the circumsporozoite protein [22], the role of the human immune system is supported by other studies documenting induced immune responses to CSP [23-26]. Taken together, the data suggest that the role of the human host's immune pressure cannot be completely excluded, even if it potentially leads to a non-protective or partially protective immune response, as might be expected in an immune-evasion strategy.

Thus, a more likely explanation would incorporate a complex interplay of a combination of factors including host-immune selection (presumably due to differential binding of parasite-derived peptides to host molecules), transmission dynamics, strong functional constraints on the protein, as well as the reproductive nature of the parasite. Which factor(s) contribute a major role at any given time and place would depend largely on a "net effect" given the local epidemiologic situation. Generally, regions of high *P. falciparum *transmission intensity correlate with high parasite prevalence and high genetic diversity of the parasite, with resultant high proportion of mixed genotypes in a given infection. This would increase the probability that a feeding mosquito obtains genetically distinct haplotypes in its blood meal, which in turn increases the probability of out-crossing in the midgut. This is consistent with data presented here considering the numerous CSP haplotypes circulating in this African endemic setting, as well as the tendency for sympatric parasites appearing to be more closely related than their allopatric counterparts (Figure 1, Table 2). In low transmission areas, it is expected that the low genetic diversity would limit the observed recombination events as detected by common algorithms, with the tendency of having a somewhat "clonal" population pattern in such low transmission areas. The finding of a predominant haplotype using larger samples in Asia e.g. the "Asia-type" (Additional file 1) and, more recently, the predominance of the 7G8 haplotype in Peru [27] would support this scenario. In short, it may be necessary to reflect these epidemiologic differences while interpreting field data on *csp *or other malaria antigen polymorphisms.

Hopefully future large-scale studies on Pf*csp *polymorphism, supplemented with well-coordinated entomologic and sero-immunological analyses, in other African endemic areas would shed more light on this issue, similar to what was recently documented for the *P. falciparum *merozoite surface protein-1 (MSP-1) in Mali [28].

## Conclusion

This study highlights an apparent discordant haplotype distribution of the T-cell epitopes of Pf-CSP with respect to age. In addition, the observed sequence polymorphism patterns were similar to that in neighbouring Gambia, but differed significantly at the sequence level from that observed in Asia. Thus, the interpretation of genetic diversity data of malaria antigen genes should probably reflect not only the antigen but also the local epidemiologic situation. In the context of the RTS,S vaccine, its future deployment in Sierra Leone is envisioned to confer similar protection in children based on extrapolation of efficacy results from The Gambia and Mozambique, because the 3D7 haplotype appears to be prevalent in this endemic setting.

## Competing interests

The authors declare that they have no competing interests.

## Authors' contributions

AJ, MJ and HM participated in the field and molecular genetic studies, AJ performed the sequence alignment and drafted the manuscript. HM and AJ conceived of the study, and participated in its design and coordination. All authors read and approved the final manuscript.

## Supplementary Material

Additional file 1 **Summary of C-terminal sequences of *P. falciparum *circumsporozoite protein of field isolates (N = 375) from Asia and Africa**. The data presented represents a comparison of haplotype (H) proportions of sequences of *P. falciparum csp *of field isolates collected from different countries in Asia and Africa. Only the region of the gene spanning the Th2R and Th3R was used (i.e. amino acid positions 326 – 394, with reference to the sequence of the 7G8 strain). Click here for file
